# Impact of Vitamin E‐Coated Membrane Hemodiafilter on Serum Albumin Redox State in the Acute Kidney Injury Pig Hemodialysis Model

**DOI:** 10.1111/aor.14982

**Published:** 2025-03-19

**Authors:** Shouichi Fujimoto, Masahide Koremoto, Shushi Yamamoto, Hiroshi Umeno, Yusuke Sano, Toshihiro Tsuruda

**Affiliations:** ^1^ M&N Collaboration Research Laboratory, Department of Medical Environment Innovation Faculty of Medicine, University of Miyazaki Miyazaki Japan; ^2^ Blood Purification Business Division Asahi Kasei Medical Co., Ltd. Tokyo Japan; ^3^ Division of Companion Animal Surgery, Department of Small Animal Clinical Sciences, School of Veterinary Medicine Rakuno Gakuen University Ebetsu Japan; ^4^ Department of Hemo‐Vascular Advanced Medicine, Cardiorenal Research Laboratory, Faculty of Medicine University of Miyazaki Miyazaki Japan; ^5^ Applied Technology Development Group, Research and Development Department Asahi Kasei Medical Co., Ltd. Fuji Shizuoka Japan

**Keywords:** acute kidney injury, hemodiafilter, oxidative stress, reduced albumin, vitamin E‐coated

## Abstract

**Background:**

Several studies have evaluated the biocompatibility of dialysis membranes. The use of vitamin E‐coated membranes has been reported multilaterally in in vitro and clinical studies. Nevertheless, the effect of vitamin E‐coated membranes on the redox state of serum albumin, which forms the largest fraction of reactive sulfhydryl groups, has not been reported.

**Methods:**

Hemodiafiltration (HDF) with and without a vitamin E‐coated hemodiafilter (V‐RA^TM^ group and ABH^TM^ groups, respectively) was performed in an acute kidney injury pig model to determine whether changes in the serum albumin, the oxidized albumin (OxiALB), and the reduced albumin (RedALB) levels differ between the two groups.

**Results:**

Analyses were conducted 22–24 times in the V‐RA^TM^ group and 16–18 times in the ABH^TM^ group, excluding missing data. The serum albumin levels decreased in both groups after nephrectomy; however, the decrease observed in the V‐RA^TM^ group was significantly lesser than that in the ABH^TM^ group. RedALB levels were significantly higher in the V‐RA^TM^ group; in contrast, OxiALB levels did not differ between the two groups. A significant positive correlation was observed between the serum albumin and RedALB levels.

**Conclusions:**

The present study demonstrated that HDF performed using a vitamin E‐coated hemodiafilter effectively minimized the reduction in serum albumin and RedALB levels compared to the vitamin E‐non‐coated hemodiafilter in an acute kidney injury pig model.

## Graphical abstract

The effect of vitamin E‐coated high‐flux hemodiafilter on the redox state of serum albumin, which forms the largest fraction of reactive sulfhydryl, has not been reported. Hemodiafiltration (HDF) with (V‐RA^TM^) and without a vitamin E‐coated hemodiafilter (ABH^TM^) was performed in an acute kidney injury pig model to determine whether changes in the serum albumin, the oxidized albumin (OxiALB), and the reduced albumin (RedALB) levels differ between the two groups. Vitamin E‐coated hemodiafilter rescued the reduction of serum albumin and RedALB in a pig model with acute kidney injury (AKI), but the vitamin E‐non‐coated hemodiafilter did not minimize and significantly decreased those when compared with baseline. HDF with the vitamin E‐coated hemodiafilter effectively minimized albumin and RedALB in a pig model with AKI.

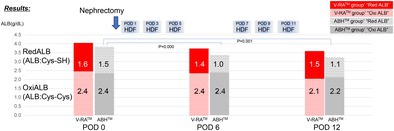

## 1 Introduction

Several in vitro studies have investigated the biocompatibility of dialysis membranes [1, 2, 3]. Vitamin E‐coated dialyzer membranes are superior to conventional dialyzer membranes in terms of controlling the progression of renal anemia and inhibiting blood coagulation during maintenance hemodialysis therapy [4, 5, 6]. However, few studies have examined the usefulness of vitamin E‐coated dialyzers on reduced albumin in the context of dialysis therapy for acute kidney injury. Patients undergoing dialysis develop oxidative stress, which generates increased levels of protein oxidation products and misfolded proteins, leading to a poor prognosis. The regulation of albumin redox is central to the mechanisms of protein homeostasis (proteostasis) and immune dysfunction (immunoproteostasis) [7, 8].

A previous study evaluating the efficacy of dialysis membranes in an in vivo pig hemodialysis model [9] revealed that pigs that underwent hemodialysis/hemodiafiltration using vitamin E‐coated membranes had lower advanced oxidation protein product levels early after commencing dialysis than those with vitamin E‐non‐coated membranes. Furthermore, reduced fibrinogen adhesion capacity to the dialysis membrane was observed in these pigs on postoperative day 1 (POD 1). Determining the redox state of serum albumin, an oxidative stress marker that forms the largest fraction of reactive sulfhydryl (free thiol groups) and acts as an effective antioxidant system in plasma [10], is difficult but essential. The level of the human non‐mercaptoalbumin (HNA) fraction (oxidative forms) in serum albumin is correlated with the risk of death from cardiovascular diseases in normoalbuminemic patients undergoing hemodialysis [11]. However, no previous studies have evaluated the serum albumin redox state in patients with acute kidney injury during hemodialysis therapy.

Therefore, this study aimed to investigate whether the serum albumin redox state, as a primary endpoint during hemodiafiltration (HDF) treatment for acute kidney injury, differs between two types of membranes (a vitamin E‐non‐coated or vitamin E‐coated membrane) using samples from a recently reported study [9].

## 2 Methods

### 2.1 Animals and Post On‐Line HDF With Pig Model

All animal experiments conducted in this study were approved by the Animal Experiment Committee at Miyazaki University (approval numbers: 2018‐017 and 2019‐035) and Asahi Kasei Medical (approval numbers: Ohito 18‐002 and Ohito 19‐010). Two groups were created based on the type of hemodiafilter (with the same membrane area and materials) used to perform HDF: the ABH^TM^ and V‐RA^TM^ groups, which used the vitamin E‐non‐coated hemodiafilter ABH^TM^‐15PA and the vitamin E‐coated hemodiafilter V‐15RA, respectively (Asahi Kasei Medical Co. Ltd., Tokyo, Japan).

### 2.2 Treatment Conditions for HDF

For HDF treatment, four pigs were assigned to the V‐RA^TM^ group, while three pigs were assigned to the ABH^TM^ group. The experiment was performed using a post‐dilution online HDF setting with the following parameters: Qb (blood flow rate) = 200 mL/min, Qd (dialysate flow rate) = 500 mL/min, and Qf (filtrate flow rate) = 50 mL/min as an additional substitute; the treatment time was 240 min per session, and six treatments were performed every other day for 2 weeks [9].

### 2.3 Preparation of OxiALB/RedALB as a Standard Reagent and Measurement of OxiALB/RedALB Levels

Supplemental Methods present the detailed methods used for the liquid chromatography‐mass spectrometry (LC/MS) analyses of OxiALB and RedALB [12, 13, 14]. Based on a pre‐examined analysis using porcine albumin as a standard reagent, the molecular weights of OxiALB and RedALB were measured and detected using approximately 66 862 and 66 743 binding cysteine types, respectively (Supplement Figure and Method S1, S2).

### 2.4 Statistical Analysis

All statistical analyses were performed using the XLSTAT ver.2018 software (Addinsoft, Paris, France). An analysis of the covariance model was performed using the day of HDF as the covariate and the measurement data as the dependent variable to evaluate the comparability of the two types of membranes for HDF (ABH^TM^ vs. V‐RA^TM^). Additionally, the Mann–Whitney U test was used to compare data acquired at the same point in both hemodiafilters. The level of statistical significance was set at 5% for all analyses (*p* < 0.05). All values are presented as the mean ± standard deviation.

## 3 Results

HDF treatment was performed for the V‐RA^TM^ group (total 24 sessions; four pigs, six sessions each) and the ABH^TM^ group (total 18 sessions; three pigs, six sessions each). Data analysis was conducted using 22–24 values for the V‐RA group and 16–18 values for the ABH group, excluding missing data.

**FIGURE 1:**
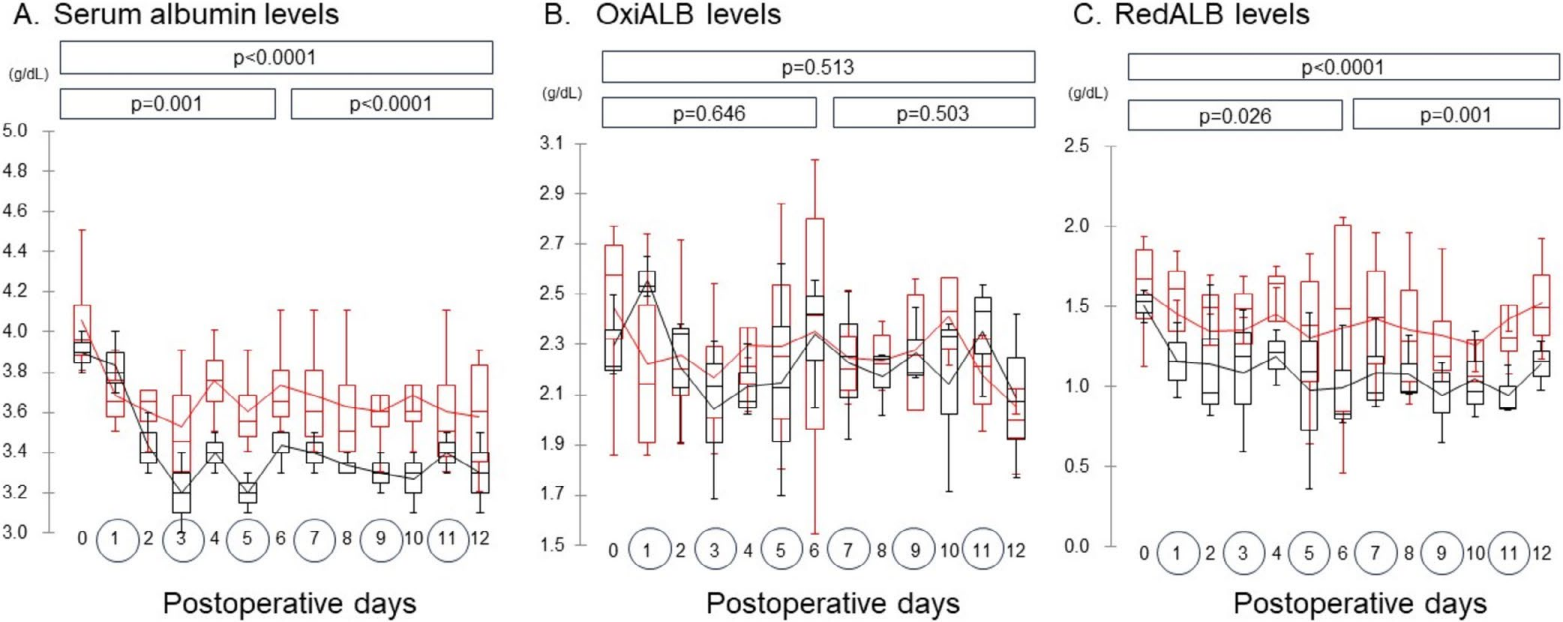
The changes in the total albumin, OxiALB, and RedALB levels, including the changes with and without hemodiafiltration, from postoperative day (POD) 0 to 12. (A) ALB: Serum total albumin. (B) OxiALB:Oxidized albumin. (C) RedALB: Serum reduced albumin. The red and black lines represent the changes in V‐RA^TM^ and ABH^TM^ groups, respectively. The line in the graph shows the average. The p‐values in the box were obtained for the analysis of the covariance model between two types of hemodiafilters from POD 0 to 6, POD 6 to 12, and POD 0 to 12. The numbers in the circles represent the day of the HDF procedure. [Color figure can be viewed at wileyonlinelibrary.com]

Figure 1 presents changes in albumin levels across PODs 1–12. Serum albumin concentration during HDF was persistently higher in the V‐RA^TM^ group than in the ABH^TM^ group, except on POD 1 (Figure 1A). No significant differences in OxiALB levels were observed between the two groups (Figure 1B). However, the RedALB levels were lower in the ABH^TM^ group (Figure 1C). A positive correlation was observed between serum albumin and RedALB levels (Pearson's correlation coefficient 0.528, *p*‐value < 0.0001).

**FIGURE 2:**
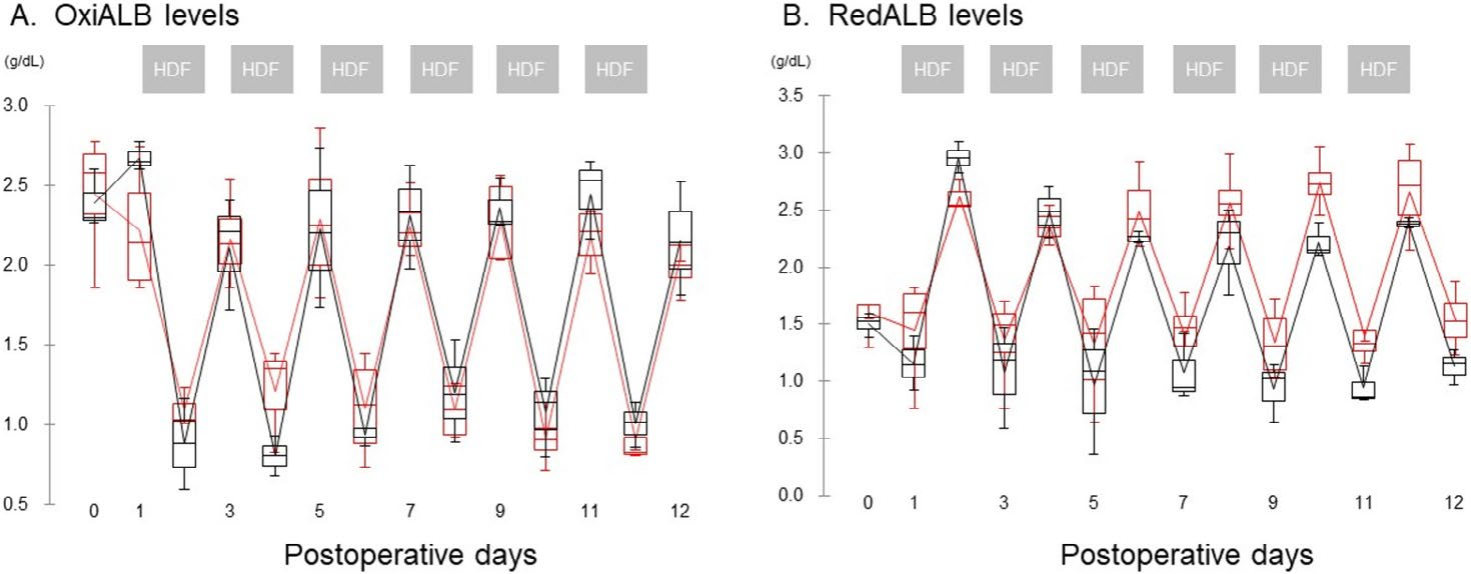
Changes in the pre‐ and post‐values of OxiALB and RedALB levels during hemodiafiltration from postoperative day (POD) 0 to 12. The red and black lines represent the changes in V‐RA^TM^ and ABH^TM^ groups, respectively. The line in the graph shows the average. [Color figure can be viewed at wileyonlinelibrary.com]

OxiALB levels decreased significantly over time during HDF (Figure 2A); however, no significant difference was observed between the two groups. In contrast, RedALB levels at the start of each HDF increased significantly compared to those at the end of each HDF in both groups (Figure 2B); however, the levels at the end of HDF in the V‐RA^TM^ group (*n* = 4) were higher than those in the ABH^TM^ group (*n* = 3) during the fourth and fifth HDF treatments (*p* = 0.057).

Table 1 shows RedALB levels before the first HDF treatment on POD1 and the changes in RedALB levels in the V‐RA^TM^ and ABH^TM^ groups during HDF using hemodiafilters. Albumin levels before the 1st HDF treatment on POD1 did not differ between the two groups. RedALB levels increased after the start of HDF treatment compared to those before HDF treatment in both groups. However, the RedALB levels in the V‐RA^TM^ group, including those at the inlet and outlet at 2 and 4 h, were significantly higher than those in the ABH group. In contrast, the degree of increase in RedALB levels before and after hemodiafiltration (inlet and outlet) at 2 and 4 h after the start of HDF treatment did not differ between the two groups. RedALB levels that were elevated at the end of HDF were lower at the start of the next HDF; the extent of the decline did not differ between the two groups (Figure 2B, Table 1).

**TABLE 1:** Comparison of averaged RedALB levels before 1st HDF treatment at POD1 and during the entire HDF treatment period between V‐RA^TM^ and ABH^TM^ groups.

	V‐RA	ABH	*p*
[Pre HDF at POD1]	1.60 ± 0.31	1.51 ± 0.11	0.857
[Averaged over HDF treatments]			
Pre HDF	1.38 ± 0.36	1.04 ± 0.31	**0.003**
2 Hr IN	1.91 ± 0.34	1.68 ± 0.25	**0.015**
2Hr OUT	2.50 ± 0.32	2.25 ± 0.22	**0.015**
2Hr changes (IN → OUT)	0.56 ± 0.31	0.55 ± 0.34	0.779
4Hr IN	2.08 ± 0.36	1.80 ± 0.25	**0.009**
4Hr OUT	2.58 ± 0.29	2.38 ± 0.31	**0.048**
4Hr changes (IN → OUT)	0.46 ± 0.27	0.54 ± 0.26	0.506
4Hr OUT → next Pre HDF	−1.12 ± 0.34	−1.29 ± 0.26	0.196

*Note:* The number of samples before 1st HDF treatment was 4 and 3 in groups V‐RA^TM^ and ABH^TM^, respectively. During the entire HDF treatment period, samples were taken before HDF treatment, as well as from all INLET/OUTLET for the 2‐ and 4‐h HDF treatments (V‐RA^TM^: *n* = 22–24; ABH^TM^: *n* = 16–18). Changes in RedALB levels after HDF and before the following HDF are also shown (V‐RA^TM^: *n* = 22–24; ABH^TM^: *n* = 16–18). Bold indicates a significant difference.

## 4 Discussion

The lower the albumin levels, the higher the risks of acute kidney injury and subsequent mortality. Notably, both albumin and its redox state levels could be crucial in the progression of kidney disease [15, 16, 17]. There is a decrease in RedALB levels, with a corresponding increase in the ratio of the oxidized form (percent of OxiALB/(OxiALB and RedALB)) in critically ill patients and post‐surgery patients [7, 8, 18, 19]. For hemodialysis patients, the human mercaptalbumin fraction (RedALB) of human albumin increased 3–5 h after starting hemodialysis and then decreased to subnormal levels [20, 21] which is consistent with the present results.

Until recently, no studies have examined redox status in acute kidney injury using a 2‐week‐long dialysis model. The current study used an in vivo pig acute kidney injury model in the V‐RA^TM^ group (vitamin E‐coated membranes) and demonstrated a lesser decline in serum albumin and RedALB levels during HDF compared to the ABH^TM^ group, although the authors hypothesized that the levels of OxiALB would decrease and that of RedALB would increase in vitamin E‐coated membranes before beginning the present research (Figure 1A,C).

Serum albumin levels are known to decrease after surgery and during systemic inflammation. The causes are thought to be hepatic reprioritization of protein synthesis (the suppression of albumin synthesis and increased synthesis acute phase protein) [11, 22], redistribution of serum proteins (an increase in capillary permeability), and an increase in protein catabolism. The decreases in the serum albumin levels immediately after bilateral nephrectomy observed in the present study may be attributed to these causes, as hepatic dysfunction was not observed in either group (Figure 1A, Table 1).

Why was the subsequent decline in serum albumin levels less in group V‐RA^TM^ than in group ABH^TM^ ? The advantage of coating dialysis membranes with vitamin E is that this molecule increases at the same time the biocompatibility of the biomaterials and the antioxidant protection of blood components [23]. RCTs on these membranes have demonstrated promising results with benefits to oxidative stress, inflammation, vascular damage, and clinical management of anemia. Meta‐analyses [24, 25], while showing the positive effect of using a vitamin E‐coated membrane on the reduction of oxidative stress biomarkers, demonstrated an improvement in the inflammatory status. Considering the above, the lower decline in serum albumin levels in group V‐RA^TM^ may be related to the increase in reduced albumin with the vitamin E‐coated membrane, resulting in the suppression of inflammation and increased synthesis of albumin.

Patients who undergo HD show higher oxidized albumin and lesser reduced albumin levels compared to healthy controls; however, the oxidized albumin fraction decreases while the reduced albumin fraction increases after HD treatment [16, 20, 26, 27]. Furthermore, these effects were significantly more pronounced in patients treated with high‐flux than low‐flux dialyzers [28, 29, 30]. In dialysis‐dependent acute renal failure, the levels of antioxidant vitamins such as α‐tocopherol were reduced and directly correlated with albumin and total antioxidant capacity of serum (ex. oxygen radical absorbance capacity: ORAC). The reduction in the ORAC was more pronounced during dialysis with cellulose acetate than with polysulfone membranes [31]. In contrast, irreversibly oxidized HNA‐2 levels show no variation during hemodialysis [20, 28] and increase over the course of plasmapheresis treatments of commercial human albumin and remain elevated 12 days after the last plasmapheresis procedure [32]. The V‐RA^TM^ and ABH^TM^ groups differed from each other in terms of the hemodiafilters used in the present study; however, the properties and area of the membranes and dialysis method were identical in both groups [9]. Additionally, previous reports comparing vitamin E‐non‐coated hemodiafilter ‘ABH^TM^’ with vitamin E‐coated hemodiafilter ‘V‐RA^TM^’ in human clinical studies reported that the effect of albumin leakage from both hemodiafilters during post‐dilution HDF is not significantly different [33]. Considering this, it is possible that in the present study, the decrease in oxidized albumin with the vitamin E‐coated polysulfone membrane was not dependent on the removal performance but was more likely due to its effects on proteostasis and immunometabolic complications.

RedALB levels increased and OxiALB levels decreased after HDF compared to before HDF treatment, respectively, and both returned to baseline levels at the next HDF treatment, suggesting that dialyzable uremic solutes may contribute to inducing intermolecular sulfhydryl‐disulfide exchange reactions. However, in the present study, there was no significant difference between the two groups in the extent of the increase in RedALB levels after HDF. Taking the above into account, the vitamin E‐coated membrane may have suppressed postoperative reduction in the synthesis ability of RedALB.

The potential limitations of this study are as follows: The authors did not compare the effects of oral Vitamin E administration with those of the dialysis membrane. Furthermore, the authors did not conduct a sufficient analysis of mortality, organ failure, and inflammatory and innate immunity. Future studies should explore these aspects to elucidate the comparative effects of the two membrane types.

In conclusion, the present study revealed that HDF using a vitamin E‐coated hemodiafilter rescued the reduction of serum albumin and increased RedALB levels in a pig model of acute kidney injury. Thus, vitamin E‐coated hemodiafilters may be useful in the HDF in postoperative acute renal failure owing to their excellent biocompatibility. Nonetheless, further studies should clarify the usefulness of vitamin E‐coated dialyzer membranes in clinical practice and investigate the mechanisms underlying changes in the serum albumin redox state.

## Author Contributions

S.F., S.Y., and H.U. contributed to the first original study design and interpretation of results (Yamamoto et al. [9]). S.F. and M.K. contributed to writing the manuscript. H.U. and Y.S. contributed to measuring the blood samples. M.K. contributed to the statistical design and statistical analysis. T.T. revised the manuscript. All authors have read and approved the final version of the manuscript.

## Conflicts of Interest

Masahide Koremoto, Hiroshi Umeno, and Yusuke Sano are employees of Asahi Kasei Medical Corporation.

## Supporting information


Data S1.

Data S2.

## References

[1] H. Kawanishi , M. Koremoto , and C. F. M. Franssen , “Clotting Propensity of Surface‐Treated Membranes in a Hemodialysis Set‐Up That Avoids Systemic Anticoagulation,” Seminars in Nephrology 43 (2023): 151482. 38262850 10.1016/j.semnephrol.2023.151482

[2] R. Takatsuji , M. Koremoto , Y. Fujimoto , Y. Saida , and Y. Hatanaka , “Flexible Inner Surface of Polysulfone Membranes Prevents Platelet Adhesive Protein Adsorption and Improves Antithrombogenicity In Vitro,” International Journal of Artificial Organs 47 (2024): 774–782. 39229822 10.1177/03913988241269465 PMC11823271

[3] H. Tsukao , K. Kokubo , H. Takahashi , et al., “Activation of Platelets Upon Contact With a Vitamin E‐Coated/Non‐Coated Surface,” Journal of Artificial Organs 16 (2013): 193–205. 23381644 10.1007/s10047-013-0686-4

[4] V. Panichi , A. Rosati , S. Paoletti , et al., “A Vitamin E‐Coated Polysulfone Membrane Reduces Serum Levels of Inflammatory Markers and Resistance to Erythropoietin‐Stimulating Agents in Hemodialysis Patients: Results of a Randomized Cross‐Over Multicenter Trial,” Blood Purification 32 (2011): 7–14. 21242686 10.1159/000321369

[5] T. Sanaka , T. Mochizuki , E. Kinugasa , et al., “Randomized Controlled Open‐Label Trial of Vitamin E‐Bonded Polysulfone Dialyzer and Erythropoiesis‐Stimulating Agent Response,” Clinical Journal of the American Society of Nephrology 8 (2013): 969–978. 23599410 10.2215/CJN.04680512 PMC3675844

[6] M. S. Islam , Z. A. Hassan , F. Chalmin , et al., “Vitamin E‐Coated and Heparin‐Coated Dialyzer Membranes for Heparin‐Free Hemodialysis: A Multicenter, Randomized, Crossover Trial,” American Journal of Kidney Diseases 68 (2016): 752–762. 27344212 10.1053/j.ajkd.2016.05.013

[7] F. Galli , D. Bartolini , and C. Ronco , “Oxidative Stress, Defective Proteostasis and Immunometabolic Complications in Critically Ill Patients,” European Journal of Clinical Investigation 54, no. 9 (2024): 14229, 10.1111/eci.14229. 38676423

[8] F. Galli , “Protein Damage and Inflammation in Uraemia and Dialysis Patients,” Nephrology, Dialysis, Transplantation 22, no. Suppl 5 (2007): v20–v36, 10.1093/ndt/gfm294. 17586842

[9] S. Yamamoto , H. Umeno , Y. Sano , et al., “A Chronic Intermittent Haemodialysis Pig Model for Functional Evaluation of Dialysis Membranes,” International Journal of Artificial Organs 47 (2024): 321–328. 38738648 10.1177/03913988241253152

[10] K. Oettl and R. E. Stauber , “Physiological and Pathological Changes in the Redox State of Human Serum Albumin Critically Influence Its Binding Properties,” British Journal of Pharmacology 151, no. 5 (2007): 580–590, 10.1038/sj.bjp.0707251. 17471184 PMC2013999

[11] P. S. Lim , Y. Jeng , M. Y. Wu , et al., “Serum Oxidized Albumin and Cardiovascular Mortality in Normoalbuminemic Hemodialysis Patients: A Cohort Study,” PLoS One 8 (2013): e70822. 23923025 10.1371/journal.pone.0070822 PMC3726598

[12] M. Domenicali , M. Baldassarre , and F. A. Giannone , “Posttranscriptional Changes of Serum Albumin: Clinical and Prognostic Significance in Hospitalized Patients With Cirrhosis,” Hepatology 60, no. 6 (2014): 1851–1860. 25048618 10.1002/hep.27322

[13] M. Naldi , F. A. Giannone , M. Baldassarre , et al., “A Fast and Validated Mass Spectrometry Method for the Evaluation of Human Serum Albumin Structural Modifications in the Clinical Field,” European Journal of Mass Spectrometry 19, no. 6 (2013): 491–496. 24378467 10.1255/ejms.1256

[14] A. Kawakami , K. Kubota , N. Yamada , et al., “Identification and Characterization of Oxidized Human Serum Albumin. A Slight Structural Change Impairs Its Ligand‐Binding and Antioxidant Functions,” FEBS Journal 273, no. 14 (2006): 3346–3357, 10.1111/j.1742-4658.2006.05341.x. 16857017

[15] C. J. Wiedermann , W. Wiedermann , and M. Joannidis , “Hypoalbuminemia and Acute Kidney Injury: A Meta‐Analysis of Observational Clinical Studies,” Intensive Care Medicine 36, no. 10 (2010): 1657–1665, 10.1007/s00134-010-1928-z. 20517593 PMC7728653

[16] M. L. Garavaglia , D. Giustarini , G. Colombo , et al., “Blood Thiol Redox State in Chronic Kidney Disease,” International Journal of Molecular Sciences 23, no. 5 (2022): 2853, 10.3390/ijms23052853. 35269995 PMC8911004

[17] S. M. Figueroa , P. Araos , J. Reyes , B. Gravez , J. Barrera‐Chimal , and C. A. Amador , “Oxidized Albumin as a Mediator of Kidney Disease,” Antioxidants 10 (2021): 404, 10.3390/antiox10030404. 33800425 PMC8000637

[18] A. Hayakawa , K. Kuwata , S. Era , et al., “Alteration of Redox State of Human Serum Albumin in Patients Under Anesthesia and Invasive Surgery,” Journal of Chromatography. B, Biomedical Sciences and Applications 698 (1997): 27–33. 9367190 10.1016/s0378-4347(97)00274-0

[19] D. Bartolini , M. A. Grignano , M. Piroddi , et al., “Induction of Vesicular Trafficking and JNK‐Mediated Apoptotic Signaling in Mononuclear Leukocytes Marks the Immuno‐Proteostasis Response to Uremic Proteins,” Blood Purification 52, no. 9–10 (2023): 737–750, 10.1159/000533309. 37703866

[20] H. Terawaki , K. Nakayama , Y. Matsuyama , et al., “Dialyzable Uremic Solutes Contribute to Enhanced Oxidation of Serum Albumin in Regular Hemodialysis Patients,” Blood Purification 25, no. 3 (2007): 274–279, 10.1159/000101986. 17460395

[21] A. Soejima , N. Matsuzawa , T. Hayashi , et al., “Alteration of Redox State of Human Serum Albumin Before and After Hemodialysis,” Blood Purification 22 (2004): 525–529. 15583477 10.1159/000082524

[22] D. C. Evans , M. R. Corkins , A. Malone , et al., “The Use of Visceral Proteins as Nutrition Markers: An ASPEN Position Paper,” Nutrition in Clinical Practice 36, no. 1 (2021): 22–28, 10.1002/ncp.10588. 33125793

[23] F. Galli , S. Rovidati , L. Chiarantini , G. Campus , F. Canestrari , and U. Buoncristiani , “Bioreactivity and Biocompatibility of a Vitamin E‐Modified Multi‐Layer Hemodialysis Filter,” Kidney International 54, no. 2 (1998): 580–589, 10.1046/j.1523-1755.1998.00021.x. 9690226

[24] S. K. Yang , L. Xiao , B. Xu , X. X. Xu , F. Y. Liu , and L. Sun , “Effects of Vitamin E‐Coated Dialyzer on Oxidative Stress and Inflammation Status in Hemodialysis Patients: A Systematic Review and Meta‐Analysis,” Renal Failure 36, no. 5 (2014): 722–731, 10.3109/0886022X.2014.890858. 24575826

[25] G. D'Arrigo , R. Baggetta , G. Tripepi , F. Galli , and D. Bolignano , “Effects of Vitamin E‐Coated Versus Conventional Membranes in Chronic Hemodialysis Patients: A Systematic Review and Meta‐Analysis,” Blood Purification 43 (2017): 101–122, 10.1159/000453444. 27960188

[26] G. Colombo , F. Reggiani , M. A. Podestà , et al., “Plasma Protein Thiolation Index (PTI) as a Biomarker of Thiol‐Specific Oxidative Stress in Haemodialyzed Patients,” Free Radical Biology & Medicine 89 (2015): 443–451, 10.1016/j.freeradbiomed.2015.08.022. 26453922

[27] M. Kohlová , C. G. Amorim , A. Araújo , A. Santos‐Silva , P. Solich , and M. C. B. S. M. Montenegro , “The Biocompatibility and Bioactivity of Hemodialysis Membranes: Their Impact in End‐Stage Renal Disease,” Journal of Artificial Organs 22 (2019): 14–28, 10.1007/s10047-018-1059-9. 30006787

[28] K. Boss , M. Paar , K. Waterstradt , et al., “Albumin Redox State of Maintenance Haemodialysis Patients Is Positively Altered After Treatment,” BMC Nephrology 24 (2023): 273, 10.1186/s12882-023-03317-9. 37723426 PMC10506191

[29] F. Galli , M. Bonomini , D. Bartolini , et al., “Vitamin E (Alpha‐Tocopherol) Metabolism and Nutrition in Chronic Kidney Disease,” Antioxidants 11, no. 5 (2022): 989, 10.3390/antiox11050989. 35624853 PMC9137556

[30] M. Piroddi , F. Pilolli , M. Aritomi , and F. Galli , “Vitamin E as a Functional and Biocompatibility Modifier of Synthetic Hemodialyzer Membranes: An Overview of the Literature on Vitamin E‐Modified Hemodialyzer Membranes,” American Journal of Nephrology 35 (2012): 559–572, 10.1159/000338807. 22677717

[31] V. S. Balakrishnan , J. Blumberg , B. J. G. Pereira , and B. L. Jaber , “Antioxidant and Oxidative Stress Indices in Dialysis‐Dependent Acute Renal Failure,” Blood Purification 21, no. 3 (2003): 213–219, 10.1159/000070692. 12784046

[32] K. Boss , M. Stettner , F. Szepanowski , et al., “Severe and Long‐Lasting Alteration of Albumin Redox State by Plasmapheresis,” Scientific Reports 12, no. 1 (2022): 12165, 10.1038/s41598-022-16452-4. 35842435 PMC9288533

[33] S. Osawa , H. Yamamoto , M. Itoh , et al., “Clinical Evaluation of Hemodiafilter V‐RA™,” Kidney and Dialysis Supplement 95 (2023): 99–102. (In Japanese).

