# Supplementary material for: Impact of Vitamin E‐Coated Membrane Hemodiafilter on Serum Albumin Redox State in the Acute Kidney Injury Pig Hemodialysis Model
Source: Artif Organs. 2025 Mar 19;49(6):1076–81. doi: 10.1111/aor.14982 (PMC12120807; doi:10.1111/aor.14982)
Supplement: Supplementary file 1 — Data S1. [file AOR-49-1076-s002.docx]

Supplemental material


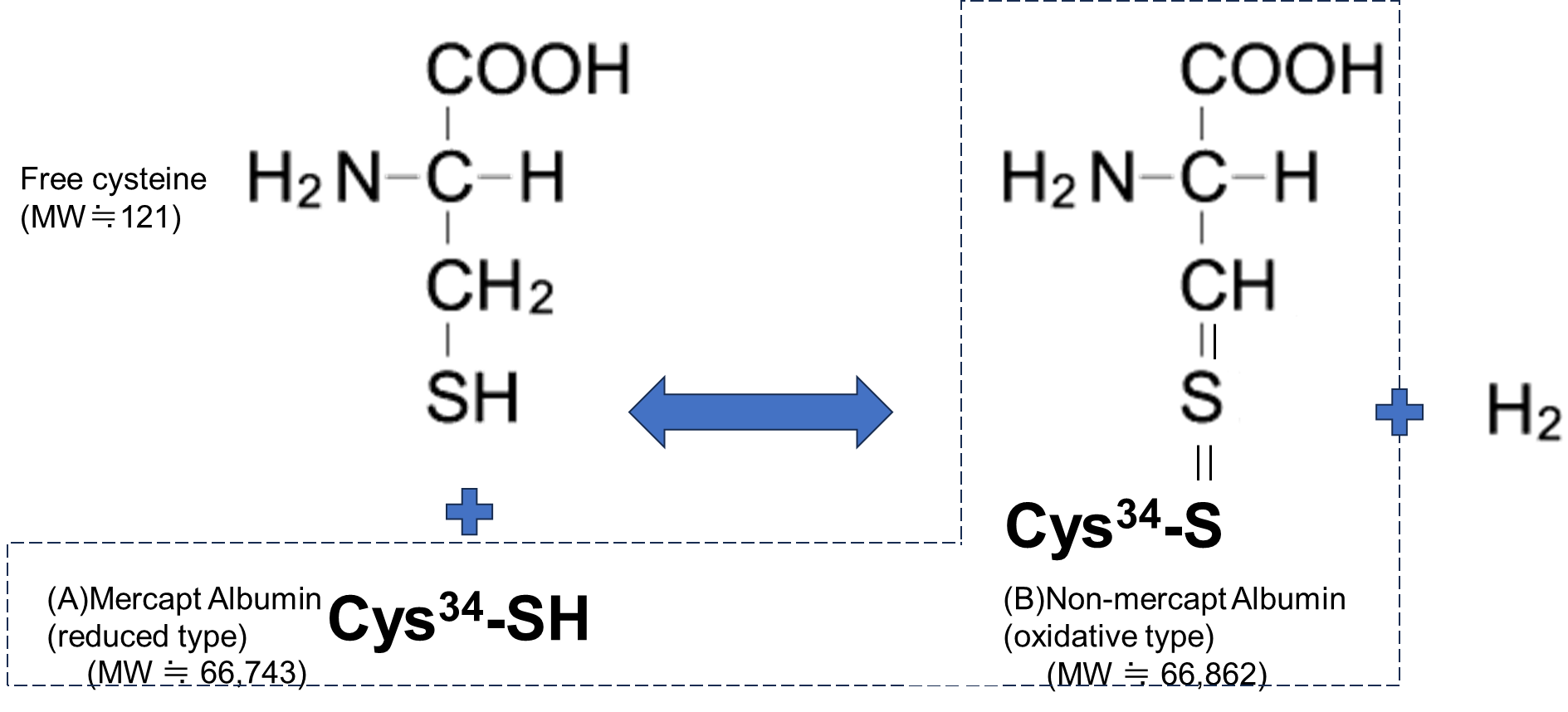


Reaction diagram of reduced and oxidized albumin. Accurate measurement was achieved through analysis using LC/MS, with oxidized/reduced albumin with N-terminal cleavage being excluded.

Supplemental Figure S1
