# Supplementary material for: Impact of Vitamin E‐Coated Membrane Hemodiafilter on Serum Albumin Redox State in the Acute Kidney Injury Pig Hemodialysis Model
Source: Artif Organs. 2025 Mar 19;49(6):1076–81. doi: 10.1111/aor.14982 (PMC12120807; doi:10.1111/aor.14982)
Supplement: Supplementary file 2 — Data S2. [file AOR-49-1076-s001.docx]

**Supplemental Methods**

**Timing of hemodiafiltration and blood sample collection**

Catheterization was performed after nephrectomy in the left lateral recumbent position. Hemodiafiltration (HDF) was performed on post-nephrectomy days 1, 3, 5, 7, 9, and 11. Blood samples collected pre-nephrectomy were designated as postoperative day (POD) 0 samples. Blood samples were collected after nephrectomy and before dialysis from the indwelling catheter after retrieval of the heparinized saline solution from the catheter (for pre-data at each POD). In addition, blood samples were collected before and after filtering through the hemodiafilter circuit at 15, 30, 60, 120, 180, and 240 minutes after commencing HDF. Blood was collected from the catheter 24 hours after the end of dialysis. A detailed description of the measurement procedures is provided. [9]

**Preparation of oxidized/reduced albumin standard reagent**

As porcine serum albumin, standard serum lyophilized powder was used (Sigma-Aldrich A1860) and adjusted at 4.0 mg/mL with 100 mmol/L sodium carbonate buffer. The diluted albumin solution (5 mL) was added to 4.5 mL of the 3 mmol/L of cysteine solution in 100 mmol/L sodium carbonate buffer and 0.5 mL of the 3 mmol/L of cystine solution in 100 mmol/L sodium carbonate buffer. The solution was mixed for 24 hours at 37 °C.

The pH of the albumin, cysteine, and cystine mixture solution were adjusted at pH 7.0 with 2 mol/L hydrochloric acid to prepare oxidized albumin. The solution was maintained at 5 °C for 24 hours, ultrafiltrated thrice with 0.1% formic acid, and adjusted at 2 mg/mL.

The albumin, cysteine, and cystine mixture solution were ultrafiltrated twice with 100 mmol/L sodium carbonate buffer and adjusted at 2 mg/mL with 0.1% formic acid to prepare reduced albumin.

**Measure equipment and condition for oxidative/reduced of albumin**

The oxidative and reduced albumin were analyzed using the Liquid Chromatograph-Mass Spectrometry (LC/MS) method.

LC-MS analyses were performed using a ACQUITY UPLC I-Class system (Waters Corporation) hyphenated to a micrOTOF-Q II ESI-Qq-TOF mass spectrometer (Bruker Daltonics Inc.).

Liquid chromatography was performed first. [11,12] Component A of the mobile phase was aqueous 0.1% formic acid. Component B was 0.1% formic acid in acetonitrile. The sample volume was adjusted to 10 μL. The analytes were separated at 80°C in a 2.1 × 50 mm BioResolve RP mAb column packed with 2.7 μm/450 Å superficially porous polyphenyl-bonded particles (Waters Corporation) at a flow rate of 0.5 mL/minute. Following sample application, components A and B underwent a gradient change: component A decreased from 75% to 45%, and component B increased from 25% to 55% between 0 to 10 minutes. Subsequently, component A increased from 45% to 95%, and component B decreased from 55% to 5% following the initial gradient change.

**Mass spectrometry analyses**

Electrospray ion source (ESI) was used as ion source for connecting LC with MS owing to its robustness and ability to analyze a various analyte, including porcine albumin. Some conditions were listed. The scan range spanned from 800 and 3000 m/z. The capillary and end plates were 4,500 and 500 volts, respectively. The nebulizer was set at 2.0 bar. The dry heater was set at 180 °C, and dry gas flowed at 8.0 L/minute.

The data pertaining to the part of the albumin peak with a S/N high ratio were extracted using DataAnalysis (Bruker Daltonics Inc.). Optimal deconvolution was performed subsequently, and the molecular weight was calculated. Albumin was identified using standard substances for oxidized albumin and reduced albumin. The abundance ratio was calculated from the ion peak intensity.

The oxidized albumin refers to albumin in which the free cysteine ​​thiol group in the albumin is bound to another cysteine, while the reduced albumin refers to albumin in which the free cysteine ​​thiol group in the albumin is in a free state. The difference in molecular weight is about 119 (supplemental Figure S1).
